# Autologous fat grafting in a case of Parry-Romberg syndrome: a case report

**DOI:** 10.1080/23320885.2026.2644772

**Published:** 2026-03-14

**Authors:** Bhakti Sarda, Sapna Nere, Bhushan Madke, Adarshlata Singh

**Affiliations:** ^a^ Department of Dermatology, Venereology & Leprosy, Datta Meghe Institute of Higher Education & Research, Wardha, India; ^b^ Department of Plastic surgery, Datta Meghe Institute of Higher Education & Research, Sawangi (Meghe), Wardha, India

**Keywords:** Parry-Romberg syndrome, progressive hemifacial atrophy, fat grafting, facial asymmetry, case report

## Abstract

Progressive hemifacial atrophy, also known as Parry-Romberg syndrome (PRS), is an uncommon condition that causes slow and progressive unilateral soft-tissue atrophy of the face. This atrophy affects the skin and subcutaneous tissue, including fat and muscle, and in some cases, may also involve the underlying bone and muscle. The exact cause of this syndrome is unknown, but several possible factors have been suggested. These include genetic predisposition, autoimmune responses, and infections or trauma. We report a case of a young female who presented with an asymptomatic one-sided atrophy of the face for the last ten years. This case highlights autologous fat grafting as a safe and effective option for restoring facial symmetry in PRS.

## Introduction

Progressive hemifacial atrophy, or Parry-Romberg Syndrome, is a unique degenerative condition marked by gradual and unilateral shrinkage of facial tissues, encompassing muscles, bones, and skin [1]. The etiology of progressive hemifacial atrophy continues to be unclear. Characteristically, the atrophy progresses slowly over many years and then becomes stable.

When development starts before the second decade of life, the underlying bone and cartilage may also be affected. A distinct boundary between normal and abnormal skin is seen, known as coup de sabre, and the affected region ranges from an isolated lesion to an extensive, significant disfigurement. Alopecia and pigmentation of the affected skin are frequently seen. Other key symptoms of this condition are enophthalmos, deviation of the mouth and nose to the afflicted side, and unilateral exposure of teeth when the lips are affected [2].

Systemic immunosuppression is used as a treatment modality to stabilise the condition. Surgery is then typically needed to regain facial symmetry [3]. Hereby, we present a case of a young female with hemifacial atrophy of the right cheek treated with fat grafting. This case report was prepared in accordance with the CARE Guidelines [4].

## Case report

A young girl in her twenties presented with complaints of depression of the right cheek for ten years. On examination, atrophy of the right cheek was noted with deviation of the tip of the nose and angle of mouth towards the affected side.

There was no significant family history, comorbidities, or past facial trauma. Psychosocial impact was evident due to facial disfigurement. MRI of the face showed reduced subcutaneous fat in the right half of the face, involving the premaxillary region and cheek. Mild bony hypoplasia of the maxilla was also noted on the affected side. Based on the history, clinical examination, and radiological findings, a diagnosis of right hemifacial atrophy was made. No significant diagnostic challenges were encountered. Mild bony hypoplasia suggested a favourable prognosis for soft tissue augmentation. The patient was taken up for autologous fat grafting.

**Figure 1.**
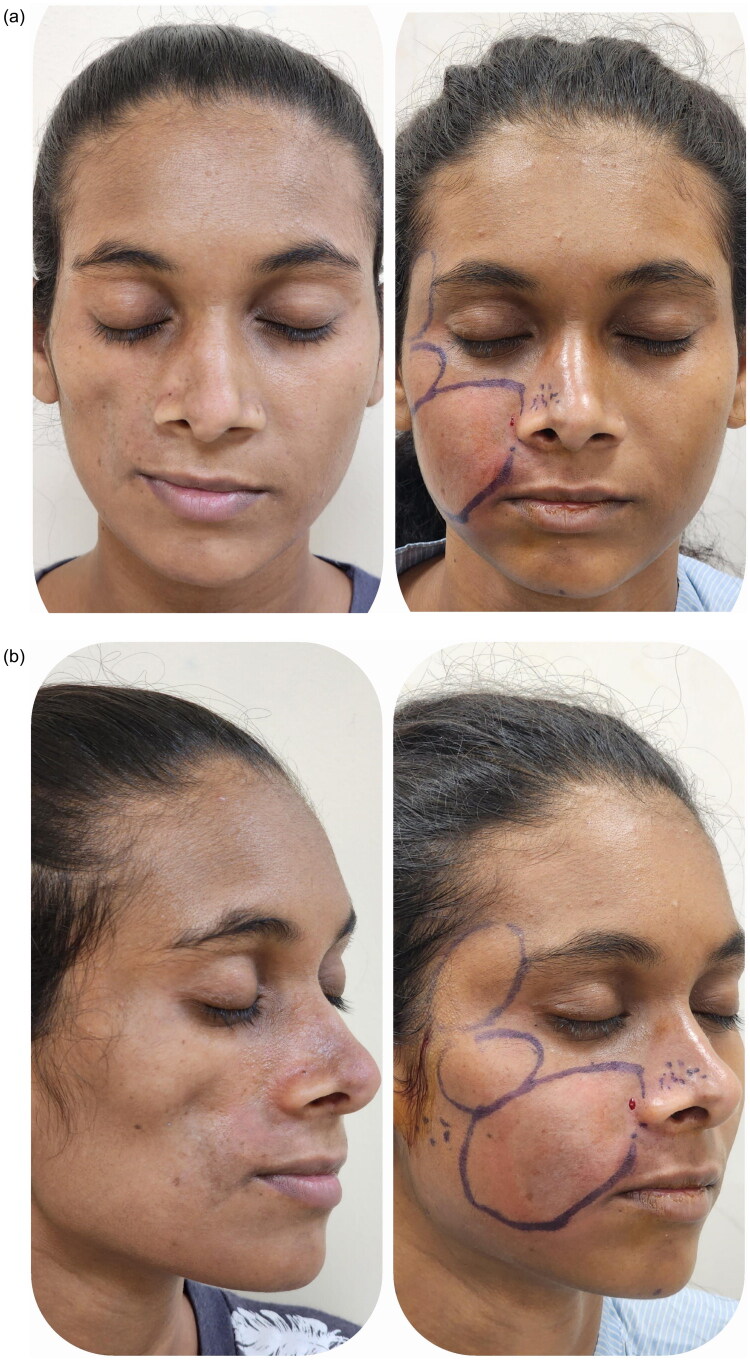
(a,b) Pre and post-operative images of 1st session of autologous fat grafting.

**Figure 2.**
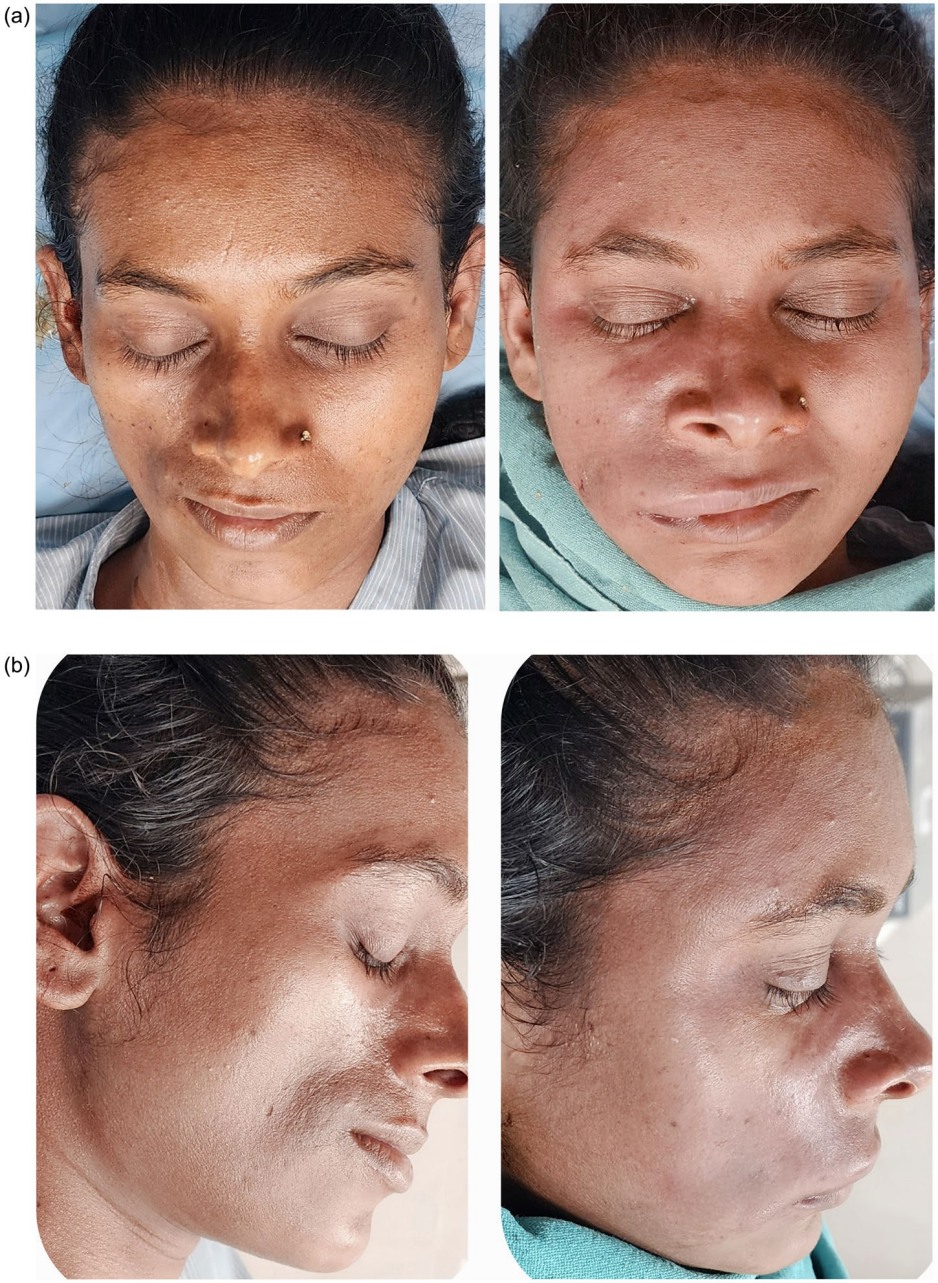
(a,b) Pre and post-operative images of 2nd session of autologous fat grafting (6 months after 1st session).

She underwent two sessions of autologous fat grafting, 40 cc (Figure 1a,b) and 30 cc (Figure 2a,b), respectively, 6 months apart. Fat was harvested from the anteromedial thigh using tumescent liposuction with low negative pressure to preserve adipocyte integrity. The lipoaspirate was processed by centrifugation at 3000 rpm for 3 min to separate the oil, aqueous, and adipocyte layers; only the purified middle layer was used for grafting. In addition, portions of the aspirate were allowed to undergo passive decantation for approximately 10 min, facilitating gentle removal of excess tumescent fluid and oil when centrifugation was not required for smaller aliquots.

**Figure 3.**
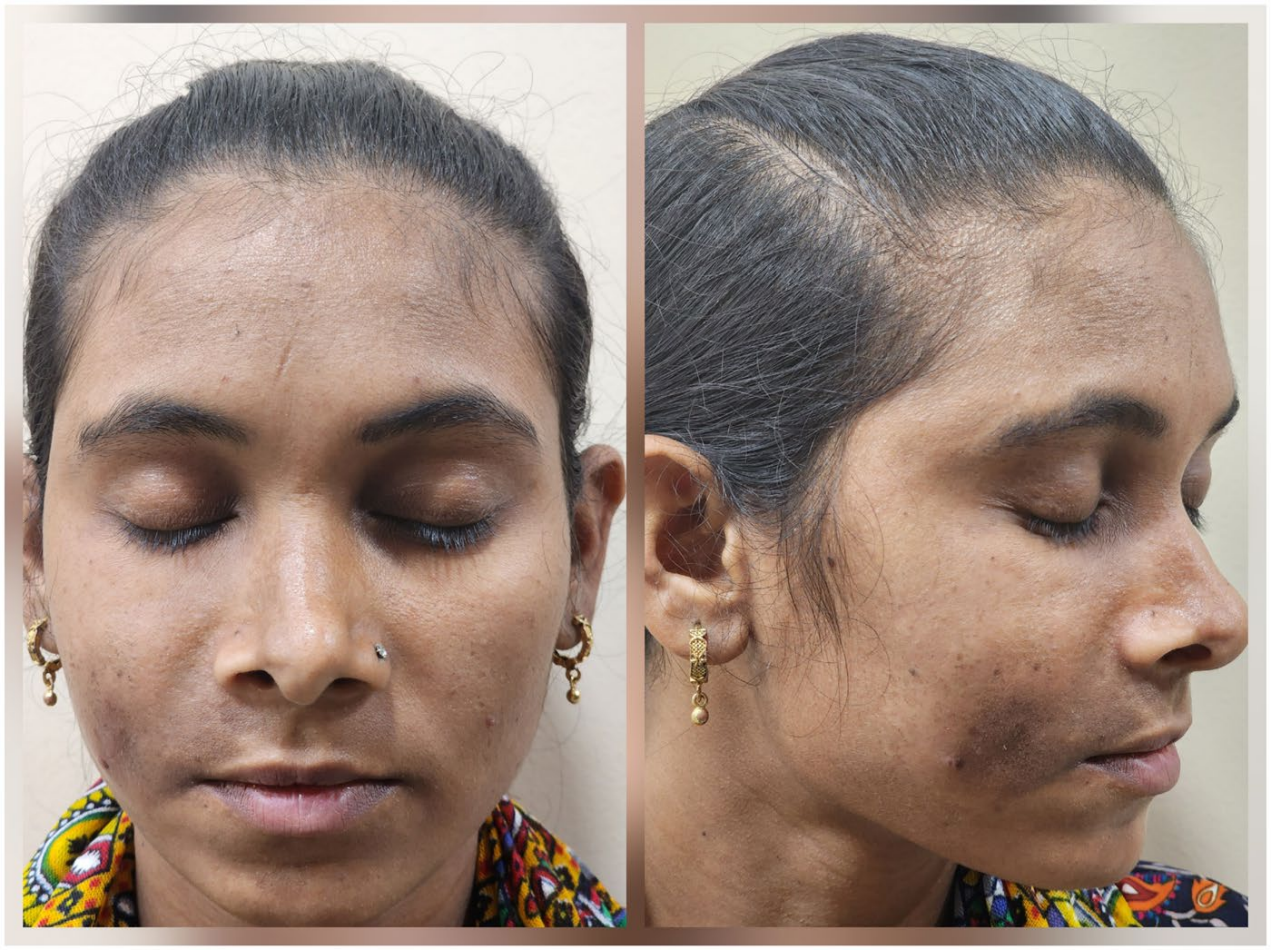
After 3 months of 2nd session of autologous fat grafting.

The process of lipofilling was multidirectional. The zygomatic arch and the body of the maxilla were injected in the subperiosteal plane, with special attention to the nasal notch area to restore projection of the alar crease. The patient adhered well to postoperative instructions. No follow-up imaging was required due to satisfactory clinical evaluation. No postoperative complications were noted, and cosmetic outcomes were satisfactory after three months of the second session (Figure 3).

## Discussion

Parry-Romberg syndrome (PRS) is a rare, acquired condition characterised by progressive hemiatrophy of the skin and soft tissue of the face, which can lead to atrophy of muscles, cartilage, and underlying bone structures in certain cases. The condition progresses at varied speeds, beginning in early childhood or puberty [5].

The cause of Parry-Romberg syndrome is unknown, while several possibilities have been presented. These include the trigeminal theory, which holds that the wasting process is caused by damage to the superior cervical ganglia, and the autoimmune hypothesis, which is based on the inflammatory alterations found at the disease’s inception and the high frequency of autoimmune antibodies detected [6]. The postulated ideas also include infection, vasculopathy, and changes in brain fat metabolism.

The loss of skin and osseocartilaginous tissues is the defining feature of this illness, which also has protean systemic signs. 20% of individuals with this condition experience a variety of neurological signs, including headache, trigeminal neuralgia, seizures, and, on rare occasions, cranial nerve palsies. In extreme cases, ocular involvement might manifest as enophthalmos, strabismus, or heterochromia. Cataract, glaucoma, uveitis, and papillitis are among the less common ocular abnormalities. Overcrowding and short crowns, and roots of teeth may be found in some people. Cognitive and behavioural issues have also been documented [7].

Differential diagnoses include hemifacial microsomia (first and second branchial arch syndrome) and its variants, such as Goldenhar syndrome, post-traumatic atrophy, and partial lipodystrophy (Barraquer-Simon syndrome). Hemifacial microsomia and Goldenhar syndrome are congenital and do not progress. Post-traumatic atrophy will have a history of trauma. Lipodystrophy is frequently bilateral and mostly affects the adipose tissue [8].

Patients with noticeable facial deformities are more likely to experience social and psychological distress. A multimodal strategy is required for the treatment of PRS. The primary goal of treatment is to halt disease activity with medical management, followed by surgical intervention to fix remaining defects.

Management is mostly cosmetic, with procedures performed once disease development has ceased and stabilised [9]. Serial fat grafting is the main treatment for individuals with minor soft-tissue atrophy, but microvascular free flaps can be used for reconstructing large-volume defects. In extreme situations, the underlying osseous structure is frequently compromised, and the transfer of vascularised tissue has traditionally been thought to be more efficient. Local flaps, such as the galea flap, are recommended to increase tissue volume and suppleness. Because microvascular free flaps can offer considerable amounts of soft tissue, they have been recommended in the latter stages of the disease.

Treatment regimens vary depending on the severity of the condition. For mild and moderate instances, soft tissue augmentation procedures are the best option for cosmetic repair. For severe cases, a combined surgical and orthodontic treatment can be performed, which includes alveolar bone augmentation, preoperative and postoperative orthodontic treatment in conjunction with orthognathic surgery, medpor filling of the zygomatic and maxillary complexes, free fat grafting, and angulus oris and lip trimming. Severe instances with substantial soft tissue and cranial bone atrophy, as well as apparent chin and occlusal plane deviation, require comprehensive therapy [10].

Autologous fat grafting is currently regarded as the preferred reconstructive technique for Parry–Romberg syndrome because it is minimally invasive, biologically compatible, and offers superior aesthetic outcomes compared to corial or microsurgical flaps. It restores facial volume by replacing lost subcutaneous fat directly, providing a more natural contour and texture (‘like replaces like’), with minimal donor-site morbidity and the flexibility for serial procedures to fine-tune results. Moreover, the presence of adipose-derived stem cells in transplanted fat promotes angiogenesis and dermal regeneration, improving skin quality and pigmentation over atrophic areas—a benefit not achieved with muscle or dermal flaps [11]. Although partial resorption can occur, the simplicity, repeatability, and regenerative potential make fat grafting particularly suitable once disease progression stabilizes [12].

Two or three stages of fat grafting may be required to get the desired outcome. The correct soft tissue replacement, which included the development of several subcutaneous and intramuscular tunnels for lipografting, guaranteed that the transplanted fat received enough blood supply. The cosmetic improvement and patient satisfaction indicate that autologous fat transplantation may be a safe approach for treating mild Parry-Romberg syndrome [13].

This case’s main strength lies in its demonstration of the potential for serial fat grafting to restore aesthetics in stable PRS. Limitations include absence of objective outcome measures and lack of long-term follow-up.

The patient expressed satisfaction with the improved symmetry and reported enhanced self-confidence post-treatment. Written informed consent was obtained from the patient for publication of this case report and accompanying images.

## Conclusion

There is little information available on the pathogenesis and treatment of PRS. Fat grafting is a simple and inexpensive treatment option for correcting disease-related facial abnormalities, even in critical cases. It is easily accessible, even in low-resource scenarios.

Good cosmetic outcomes can be achieved with fat grafting alone, even in severe cases. When given at the appropriate anatomical layer, it can produce good outcomes, including the repair of bony projection loss. Autologous fat not only serves as a pliable filler, allowing for massive soft tissue reconstructions, but it also has regenerative potential due to its high concentration of mesenchymal stem cells. Fat grafting has been proven to enhance functional and aesthetic results in individuals with PRS.

## Limitations

3D volumetric analysis could not be done due to a lack of facilities and financial constraints on the patient’s part.

## Supplementary Material

Figure legends.docx
