# Supplementary material for: Autologous fat grafting in a case of Parry-Romberg syndrome: a case report
Source: Case Reports Plast Surg Hand Surg. 2026 Mar 14;13(1):2644772. doi: 10.1080/23320885.2026.2644772 (PMC12990273; doi:10.1080/23320885.2026.2644772)
Supplement: Figure legends.docx [file ICRP_A_2644772_SM9362.docx]

Figure 1a: Pre and post-operative images of 1st session of autologous fat grafting

Figure 1b: Pre and post-operative images of 1st session of autologous fat grafting

Figure 2a: Pre and post-operative images of 2nd session of autologous fat grafting (6 months after 1st session)

Figure 2b: Pre and post-operative images of 2nd session of autologous fat grafting (6 months after 1st session)

Figure 3: After 3 months of 2nd session of autologous fat grafting
